# Comparisons of outcomes between ProKnife injection endoscopic submucosal dissection and conventional endoscopic submucosal dissection for large gastric lesions in ex vivo porcine model study: A randomized controlled trial

**DOI:** 10.1002/deo2.91

**Published:** 2022-01-26

**Authors:** Mitsuru Esaki, Eikichi Ihara, Misato Esaki, Kei Nishioka, Yusuke Kimura, Yoshitaka Hata, Hirotaka Tsuru, Masafumi Wada, Yosuke Minoda, Xiaopeng Bai, Yoshihisa Shoguchi, Takayuki Nasu, Shuzaburo Nagatomo, Kazumasa Muta, Haruei Ogino, Yoshihiro Ogawa

**Affiliations:** ^1^ Department of Medicine and Bioregulatory Science Graduate School of Medical Sciences Kyushu University Fukuoka Japan; ^2^ Department of Medicine Nihon University School of Medicine Tokyo Japan; ^3^ Department of Gastroenterology and Metabolism Graduate School of Medical Sciences Kyushu University Fukuoka Japan; ^4^ Clinical Education Center Kyushu University Hospital Fukuoka Japan; ^5^ Muta Hospital Fukuoka Japan

**Keywords:** endoscopic submucosal dissection, ex vivo porcine model, gastric lesions, predictive factors, ProKnife

## Abstract

**Objective:**

To compare treatment outcomes between injection endoscopic submucosal dissection using ProKnife (P‐ESD) and conventional ESD (C‐ESD) for gastric lesions.

**Methods:**

In this randomized controlled trial, we compared treatment outcomes of P‐ESD and C‐ESD for simulated gastric lesions ≥3 cm in resected porcine stomachs. Predictive factors associated with ESD difficulties were investigated using logistic regression analysis.

**Results:**

Seventy lesions were screened; however, two lesions were excluded. A total of 12 endoscopists performed 68 ESDs: 34 P‐ESDs and 34 C‐ESDs. The ESD procedure time of P‐ESD (36.3 [28.4–46.8] min) was significantly shorter than that of C‐ESD (46 [36.4–64.6] min; *p* = 0.0014). The technical success rates did not differ between the P‐ESD and C‐ESD groups (en bloc resection rate, 100% in both groups; complete resection rate, 94.1% and 85.3%, respectively; *p* = 0.23). The number of injections during P‐ESD (7.5 [6–10] times) was significantly higher than during C‐ESD (4 [3–5] times; *p* < 0.001), but the total volume of injected solution during P‐ESD (20 [16–26.3] ml) was significantly smaller than during C‐ESD (27.5 [20–31.5] ml; *p* = 0.0019). In multivariate analysis, less ESD experience (odds ratio [OR], 3.9) and selection of C‐ESD as the ESD method (OR, 3.8) were independent predictive factors associated with ESD difficulties.

**Conclusions:**

Compared with C‐ESD, P‐ESD had a shorter procedure time but also allowed for notable technical success and safety.

## INTRODUCTION

Endoscopic resection is an accepted standard local treatment for early gastric neoplasms because of its minimal invasiveness compared with surgery.^1^, ^2^ Although Endoscopic mucosal resection (EMR) is technically limited for large or ulcerative lesions, endoscopic submucosal dissection (ESD) has enabled en bloc and even R0 resection in such cases. ESD has been applied to gastric cancer, considering its technical success.^3^ However, to perform ESD, endoscopists must be highly skilled in manipulating endoscopic devices, including knives.^4^ Sometimes, endoscopists encounter long procedure times or intraoperative perforation during ESD. Large lesions, ulcerative lesions, undifferentiated cancers, and lesions in upper locations are considered predictive factors associated with ESD difficulties.^5^, ^6^ Novel methods and devices have been developed to overcome these difficulties.^7^, ^8^, ^9^

To perform ESD safely, high‐viscosity solutions like hyaluronic acid are injected locally into the submucosal layer using an injection needle before the mucosal incision.^10^, ^11^, ^12^ Thickening of the submucosa by local injection prevents thermal denaturation and direct damage to the muscle layer, thereby avoiding perforation. If the thickness of the submucosal layer becomes insufficient during ESD, an additional solution can be injected. Reinjection requires device replacement to resume incision or dissection, which increases the procedure time. Additionally, the effect of lifting the submucosal layer may gradually lessen before the procedure is resumed.

A novel endoscopic device called ProKnife (ORISE ProKnife; Boston Scientific, MA, USA) has recently been invented in the USA and is now available in Australia and Japan.^13^ ProKnife comprises an electrosurgical needle‐type knife with needle injection function. The injection lumen diameter of the electrode is large (0.3 mm), twice that of other needle‐type knives currently in use, including the Hybrid knife (ERBE Elektromedizin, GmbH, Tübingen, Germany). ProKnife enables rapid injection without device replacement, even with high‐viscosity solutions. Therefore, ProKnife ESD (P‐ESD) may improve treatment outcomes in difficult ESD cases. However, its efficacy and safety for gastric lesions compared with conventional ESD (C‐ESD) has not been determined. This randomized controlled trial (RCT) aimed to compare the efficacy and safety of P‐ESD and C‐ESD for simulated large gastric lesions in an ex vivo porcine model.

## METHODS

### Study design and model

This single‐center, prospective, parallel, open‐label, randomized controlled, superiority trial compared the treatment outcomes of P‐ESD and C‐ESD using ex vivo porcine models. The study was conducted from May to July 2021 at Kyushu University. Twelve endoscopists participated as ESD operators. Approval of the study protocol by the institutional ethical committee and informed consent were waived because the study involved an ex vivo porcine model and not human subjects. This study was conducted in accordance with the guidelines the Animal Research Reporting in vivo Experiments as much as possible although it was an ex vivo animal model study.

Ex vivo porcine models for ESD were made from resected porcine stomachs obtained from the local slaughterhouse. Simulated lesions ≥3 cm in diameter were created by marking dots with the electrosurgical knife, which has been reported to be technically difficult.^5^, ^6^, ^14^ The sizes of simulated lesions were measured endoscopically with an endoscopic measuring device (M2‐3U; Olympus, Tokyo, Japan). The greater and lesser curvature of the stomach was set to be the lower and upper sides of gravity, respectively. Lesions continuous to the pylorus or fossa were not used because the lower esophagus was connected to the guide tube and the duodenum just outside the pyloric ring was ligated, which could affect treatment outcomes.

### Randomization

Endoscopists had 24‐h access to a web‐based central registration and randomization system. Eligible lesions were registered and randomly assigned (1:1) to the P‐ESD or C‐ESD group. Randomization was performed using dynamic balancing, which uses the minimization method by lesion location (upper or middle‐third of the stomach vs. lower‐third of the stomach), lesion position (greater curvature vs. others), and operator's ESD experience (0–49 vs. ≥50 cases). Endoscopists were not blinded to the allocated treatment group. The locations (upper, middle, or lower third of the stomach) and positions (lesser curvature, greater curvature, anterior wall, or posterior wall) of the lesions were classified based on the current Japanese classification of gastric carcinoma.^15^

### Intervention

**FIGURE 1:**
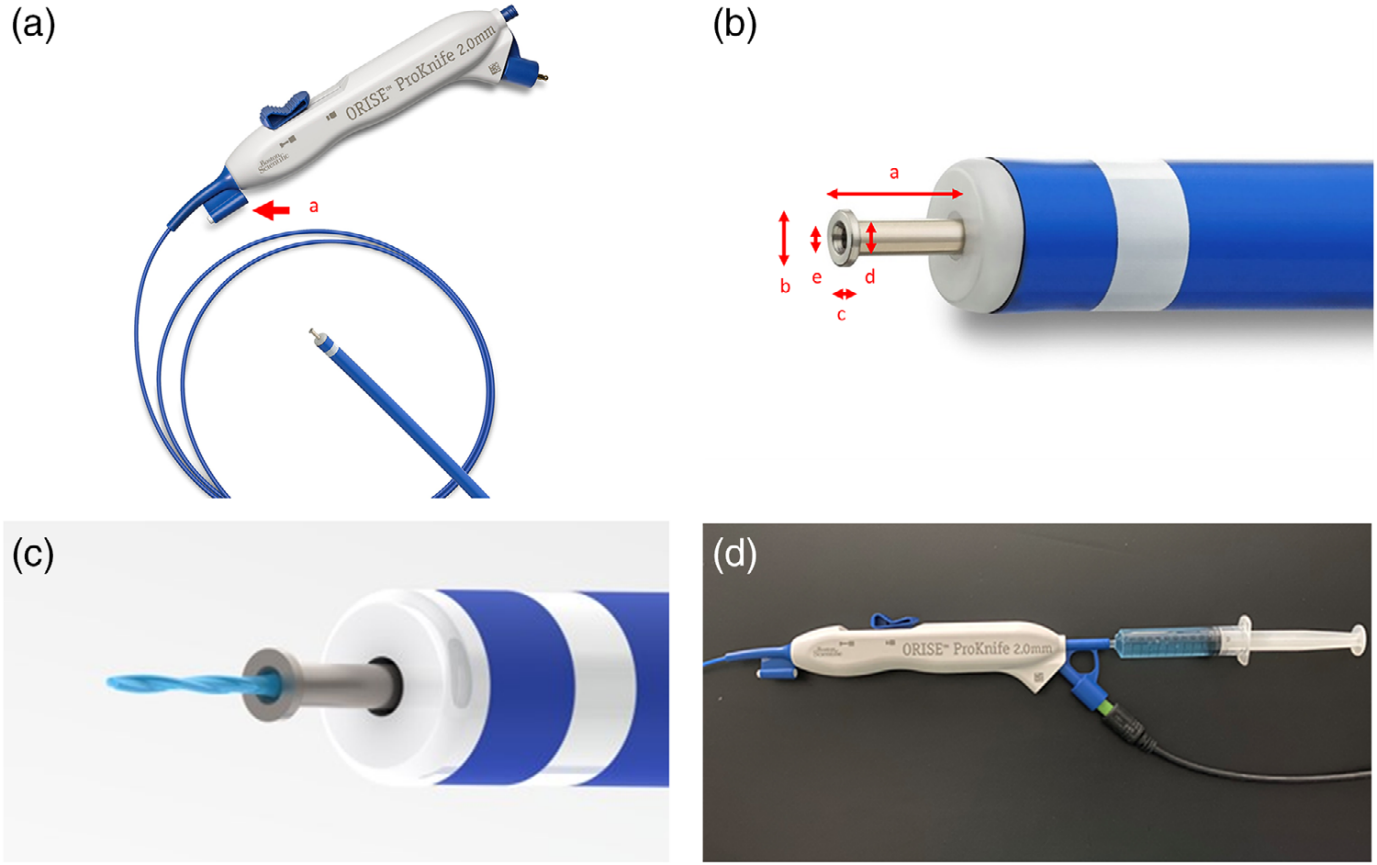
Images of ProKnife (ORISE ProKnife; Boston Scientific, Tokyo, Japan). (a) Whole image of ProKnife. Cleaning tool attached to the device (arrow a). (b) The distal tip of ProKnife. The distal tip has a 2.0 mm length (arrow a). T‐shaped tip has a 0.81 mm diameter (arrow b) and 0.15 mm thickness (arrow c). Shaft has 0.5 mm diameter (arrow d). Lumen has 0.3 mm diameter (arrow e). (c) Injection function of ProKnife. (d) ProKnife connected to the syringe containing hyaluronic acid

All procedures were performed by the participating endoscopists. All endoscopists had experience with >1000 upper or lower gastrointestinal endoscopies. ESD procedures were performed with upper gastrointestinal endoscopes (GIF‐H260; Olympus) attached to disposable hoods (Elastic Touch; Top, Tokyo, Japan). ESG‐100 (Olympus) was used as the electrosurgical unit (cutting mode, pulse cut first mode, 80 W; coagulation mode, forced Coag2, 80 W). ProKnife (Figure 1a–c) with a 2.0 mm tip length was selected and a 25‐gauge (G) injection needle (SuperGrip, Top) was used in both groups. The water‐jet system was not used in any procedures.

**FIGURE 2:**
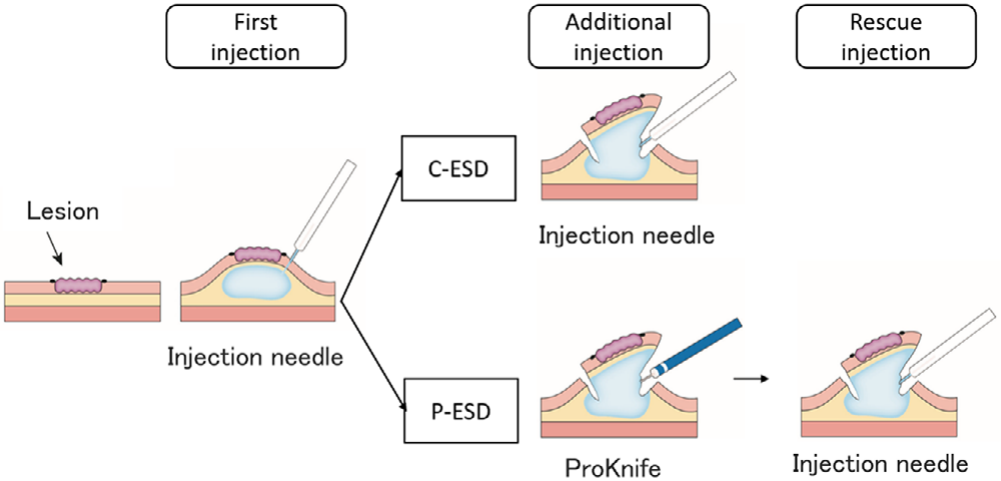
Schematic of the protocol for the injection procedures. C‐ESD, conventional endoscopic submucosal dissection; P‐ESD, ProKnife injection ESD

ESD for gastric lesions has previously been described in detail.^16^, ^17^, ^18^ Briefly, a high‐viscosity solution was injected into the submucosal layer around the markings before circumferential incision and submucosal dissection were performed. Partial submucosal dissection was allowed prior to completion of a circumferential mucosal incision. Additional injections could be performed whenever needed. ESD was finalized by completing a submucosal dissection. We used a 0.4% hyaluronic acid solution with a small amount of indigo carmine in all injections. Firstly, 2 ml of the hyaluronic acid solution was injected into the submucosal layer using a 25‐G injection needle in both groups. In P‐ESD, any additional injection was performed using ProKnife (Figure 1d). In C‐ESD, any additional injection was conducted using another 25‐G injection needle. A small incision was sometimes required to allow the tip of ProKnife to penetrate the submucosal layer because its tip is not as sharp as an injection needle. If sufficient elevation of the submucosal layer could not be achieved by injection through ProKnife in P‐ESD, rescue injection using a 25‐G injection needle was allowed. The number of injections and total volumes of injection solution were recorded. Injections into multiple sites during one interruption of cutting were counted as one. The protocol for the injection procedures is shown in Figure 2. The planned use of ESD‐assisting techniques was restricted, including traction, pocket creation, and tunnel creation. A temporary change of operators or use of ESD‐assisting techniques was allowed as rescue, if necessary. For example, when the ESD procedure time exceeded 60 min or intraoperative perforation occurred. Operator change was considered prior to the use of ESD‐assisting techniques. The resected specimens were pinned to a plastic plate for evaluation. The long and short‐axis diameters of resected specimens were measured using a scale. Additionally, all markings were checked to ensure that they remained within the resected specimens. The circumferences (mm) and resected areas (mm^2^) were obtained using the long and short axes of the resected specimens.

### Outcomes

The primary outcome was ESD procedure time, defined as the total time from mucosal incision to complete retrieval of the lesion, including mucosal incision, submucosal dissection, and additional injection. The secondary outcomes were as follows: the time and speed of mucosal incision, time and speed of submucosal dissection, en bloc resection rate, complete resection rate, the total volume of high‐viscosity solution used, number of injections (total/using the 25‐G injection needle/using ProKnife), and rate of operator change and traction usage. En bloc resection was defined as non‐piecemeal lesion resection. Complete resection was defined as en bloc resection with all marking dots confirmed within the resected specimen. Intraoperative perforation was determined when an immediately recognizable hole was observed in the stomach wall. The speed of mucosal incision was calculated as the circumference of the resected specimen/incision time (mm/min). The speed of submucosal dissection was calculated as the area of resected specimen/dissection time (mm^2^/min). Furthermore, we estimated the predictive factors associated with ESD difficulties, including long procedure time, operator change, and intraoperative perforation. Covariate factors were defined as lesion location, position, estimated size, operator skill, and ESD method. Long procedure time was defined as ≥50 min based on the third quartile (75th percentile) of ESD procedure time. The estimated tumor size was categorized as the third quartile (75th percentile).

### Statistical analysis

As a pilot study, we performed 10 C‐ESD procedures for the same model of this study (Table S1). The median ESD procedure time was 43 min with a variance of 734.9.

Previous studies reported that ESD with other water‐jet function endo‐knives achieved a 25%–50% reduction of procedure time compared with C‐ESD.^19^, ^20^, ^21^ We hypothesized that a 30% reduction in ESD procedure time would indicate a clinically relevant improvement of P‐ESD over C‐ESD. Therefore, the median procedure time for P‐ESD was assumed to be 30.1 min, with a reduction of 30% (12.9 min) from the median procedure time for C‐ESD. The variance was assumed to be equal for both procedures. The distribution of procedure time was expected to be skewed to the right. Therefore, the required sample size was calculated by log‐transforming the data before performing a *t*‐test. Assuming that the transformed procedure time would follow a log‐normal distribution, the mean values after log transformation were 3.7612 for C‐ESD and 3.4045 for P‐ESD, with a common standard deviation of 0.5157.^22^ The required sample size of 68 cases (34 cases per group) was calculated to ensure 80% power for a two‐sided significance level of 0.05.

Continuous variables are presented as medians with interquartile ranges and were analyzed using the Mann–Whitney U test. ESD procedure time was analyzed using the *t*‐test after log transformation. Categorical variables are presented as frequencies with percentages and were analyzed using the chi‐square or Fisher test. Multivariate analysis was performed using logistic regression analysis. *p*‐values <0.05 were considered statistically significant. All statistical analyses were performed using JMP 15.0 software (SAS Institute, Cary, NC, USA).

## RESULTS

### Included lesions

**FIGURE 3:**
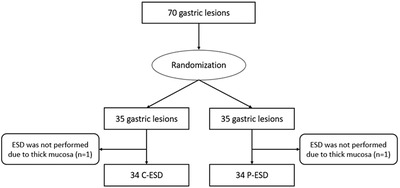
Flow chart of enrolled lesions in this study. C‐ESD, conventional endoscopic submucosal dissection; P‐ESD, ProKnife injection ESD

A flow chart of lesion inclusion is shown in Figure 3. Seventy ESD cases of simulated gastric lesions were screened. Two cases were excluded because the lesion mucosa was deemed too thick to be incised. Finally, 68 cases of ESD were included and randomized (P‐ESD, 34 cases; C‐ESD, 34 cases).

### Lesion characteristics

Lesion characteristics are shown in Table 1. No significant difference was noted in characteristics between the P‐ESD and C‐ESD groups. The details about the number of ESD procedures, the lesion characteristics, and the selected ESD method for each operator are shown in Table S2.

**TABLE 1:** Background characteristics of enrolled lesions

	**All**	**C‐ESD**	**P‐ESD**	
	***n* = 70**	***n* = 35**	***n* = 35**	***p*‐value**
Tumor location				0.81
Upper or Middle	37 (53)	18 (51)	19 (54)	
Lower	33 (47)	17 (49)	16 (46)	
Tumor position				1
L or A or P	40 (57)	20 (57)	20 (57)	
G	30 (43)	15 (43)	15 (43)	
Estimated long axis diameter, mm	35 (35–40)	35 (32–40)	35 (35–40)	0.42
Estimated short axis diameter, mm	30 (25–35)	30 (25–32)	30 (25–35)	0.76
Estimated size (mm^2^)	824.3 (628–961.6)	824.3 (686.9–942)	824.3 (628–961.6)	0.73
Operator skill				0.81
0–49 cases of ESD	39 (56)	20 (57)	19 (54)	
≥50 cases of ESD	31 (44)	15 (43)	16 (46)	

Values are presented as median (interquartile range) or number (frequency [%])

Abbreviations: A, anterior wall; C‐ESD, conventional ESD; ESD, endoscopic submucosal dissection; G, greater curvature; L, lessor curvature; P, posterior wall; P‐ESD, ProKnife injection ESD.

### Treatment outcomes

The treatment outcomes are shown in Table 2. The procedure time of P‐ESD (36.3 [28.4–46.8] min) was significantly shorter than that of C‐ESD (46 [36.4–64.6] min; *p* = 0.0014). The number of P‐ESD injections (7.5 [6–10] times) was significantly higher than that of C‐ESD (4 [3–5] times; *p* < 0.001). However, the total volume of injection solution in P‐ESD (20 [16–26.3] ml) was significantly smaller than that of C‐ESD (27.5 [20–31.5] ml; p = 0.0019). Compared with in C‐ESD, the time and speed of mucosal incision and submucosal dissection in P‐ESD were significantly shorter and quicker, respectively. Other outcomes were not significantly different between the two groups.

**TABLE 2:** Treatment outcomes of conventional endoscopic submucosal dissection (C‐ESD) and ProKnife injection ESD (P‐ESD)

	**All**	**C‐ESD**	**P‐ESD**	***p*‐value**
	**n = 68**	**n = 34**	**n = 34**	
ESD procedure				<0.001
Time (min)	40 (33–51)	46 (36.4–64.6)	36.3 (28.4–46.8)	
Log‐transformed time (min)	3.73 (0.37)	3.88 (0.36)	3.59 (0.32)	
En bloc resection, *n* (%)	68 (100)	34 (100)	34 (100)	‐
Complete resection, *n* (%)	61 (89.7)	29 (85.3)	32 (94.1)	0.23
Perforation, *n* (%)	3 (4.4)	1 (2.9)	2 (5.9)	0.55
Operator's change, *n* (%)	9 (13.2)	5 (14.7)	4 (11.8)	0.72
Assist‐technique usage	0 (0)	0 (0)	0 (0)	‐
Number of injections	5.5 (4–9)	4 (3–5)	7.5 (6–10)	<0.001
Rescue injection, *n* (%)	‐	‐	7 (20.6)	‐
Total volume of injective solutions (ml)	23 (18.3–29)	27.5 (20–31.5)	20 (16–26.3)	0.0019
Circumferential length (mm)	113.9 (103.0–127.6)	112.2 (102.2–128.4)	114.2 (103.3–128.0)	0.60
Resected specimen size (mm^2^)	989.1 (830.5–1271.7)	971.8 (821.5–1285.8)	997.0 (835.2–1278.8)	0.71
Mucosal incision				
Time (min)	17.5 (13.1–22.0)	21.3 (16–25.4)	16 (12–19)	0.0014
Speed (mm/min)	6.6 (5.2–8.6)	5.4 (4.5–7.4)	7.4 (6.3–9.4)	0.0011
Submucosal dissection				
Time (min)	22.5 (16.6–30.9)	25.3 (18.9–41.4)	19.5 (16–28.5)	0.023
Speed (mm^2^/min)	41.5 (32.7–60.4)	36.4 (27.2–50.9)	49.9 (38.3–63.1)	0.006

Values are presented as median (interquartile range) or frequency (%).

Log‐transformed time are presented as mean (standard deviation).

Abbreviations: C‐ESD, conventional ESD; P‐ESD, ProKnife injection ESD.

### Predictive factors associated with difficulties of endoscopic submucosal dissection

Results of the univariate and multivariate analyses of predictive factors associated with ESD difficulties are shown in Table 3. In multivariate analysis, experience with 0–49 cases of ESD (odds ratio [OR], 3.9; 95% confidence interval [CI], 1.2–13.4) and C‐ESD as the ESD method (OR, 3.8; 95% CI, 1.1–13.0) were independent predictive factors associated with ESD difficulties.

**TABLE 3:** Univariate and Multivariate analysis for the factors associated with the difficulty of endoscopic submucosal dissection (ESD) including long procedure time (≥50 min), operator change, and perforation

			**Univariate**			**Multivariate**		
	**No. of Patients**	**No. of events**	**OR**	**95% CI**	***p*‐value**	**OR**	**95% CI**	***p*‐value**
Location								
Lower	32	7	1	Ref	0.053	1	Ref	0.051
Upper or middle	36	16	2.9	0.98–8.3		3.34	0.96–11.7	
Position								
L or A or P	39	10	1	Ref	0.10	1	Ref	0.10
G	29	13	2.4	0.84–6.6		2.7	0.82–8.8	
Estimated size								
<950 mm^2^	48	14	1	Ref	0.21	1	Ref	0.27
≥950 mm^2^	20	9	2.0	0.68–5.8		2.1	0.56–7.9	
Operator skill								
≥50 cases	31	6	1	Ref	0.024	1	Ref	0.022^a^
0–49 cases	37	17	3.5	1.2–10.6		3.9	1.16–13.4	
Method								
P‐ESD	33	8	1	Ref	0.076	1	Ref	0.029^a^
C‐ESD	33	15	2.6	0.90–7.3		3.8	1.1–13.0	

^a^ significant value

Abbreviations: A, anterior wall; C‐ESD, conventional ESD; CI, confidence interval; ESD, endoscopic submucosal dissection; G, greater curvature; L, lessor curvature; OR, odds ratio; P, posterior wall; P‐ESD, ProKnife injection ESD.

## DISCUSSION

This is the first study to evaluate the efficacy and safety of P‐ESD compared with C‐ESD in an ex vivo porcine stomach model. Our study revealed that compared with C‐ESD, P‐ESD significantly shortened the ESD procedure time and contributed to a reduction in the total volume of injection solution. Furthermore, selection of C‐ESD and low operator skill were independent predictive factors associated with ESD difficulties.

During the development of the ESD procedure, the use of several electrosurgical knives with water‐jet injection function has been proposed for gastrointestinal tumors. By this method, local injection of saline can be performed using the knife as required at any time during mucosal incision or submucosal dissection.^19^, ^20^, ^21^, ^23^ Previous RCTs revealed the effectiveness of ESD using electrosurgical knives with a conventional water‐jet injection function compared to C‐ESD for gastrointestinal tumors.^19^ The use of such devices eliminates the time wasted during device replacement for injection. Although it is technically possible to inject high‐viscosity solutions through these knives, a sufficient flow cannot be achieved due to their lumen diameter which is narrower than that of an injection needle. In contrast, ProKnife has a wide needle‐lumen and can be used to efficiently inject high‐viscosity solutions, suggesting that ProKnife could replace the injection needle. However, there is currently a lack of evidence regarding P‐ESD. Therefore, we designed an RCT of C‐ESD and P‐ESD using *ex vivo* porcine models created from resected porcine stomachs, which could precede a similar study in humans if deemed ethically appropriate. We considered that P‐ESD could reduce both ESD procedure time and its associated difficulties. Thus, we included lesions ≥3 cm in this study to simulate technically challenging lesions for ESD.^5^, ^6^, ^14^

The primary outcome, ESD procedure time, was significantly shortened by P‐ESD (21% reduction in median time) compared with C‐ESD. Several factors may have contributed to the reduction of the ESD procedure time. First, P‐ESD allowed 80% of the operators to complete ESD without device replacement during treatment. In contrast, in C‐ESD, device replacement occurred a median of six times with three additional injections of the viscous solution following the first injection being required. Second, P‐ESD allowed operators to perform timely injections of viscous solution only to the target areas when required. Third, maximal lifting effects of the target area could be achieved since either incision or dissection could be performed immediately after injection of high‐viscosity solution and before it spread to surrounding tissues. Furthermore, the procedure times of mucosal incision or submucosal dissection in P‐ESD were also significantly shorter than those of C‐ESD. Lesions in this study were large (≥3 cm diameter) and most required additional injections during the submucosal dissection and circumferential incision phases. As a result, P‐ESD contributed to a significant reduction of procedure time in both phases.

Timely injection to the target area also contributed to the reduction of total solution injection volumes. Since high‐viscosity solutions like hyaluronic acid are costly, the reduction in total volume is directly linked to a reduction in total medical costs. In our study, the first injection was made using a 25‐G injection needle in both groups because the first mucosal pre‐cut with ProKnife was considered a risk for perforation. However, we have previously reported that pre‐cutting using another endo‐knife with conventional injection functionality could be achieved without perforation if done carefully, even without an injected solution.^24^, ^25^, ^26^ In the future, it is possible that all ESD procedures may be completed using a single device like ProKnife, without the need for an injection needle.

The ESD method and operator experience were independent predictive factors associated with ESD difficulties. High endoscopic skills were required to complete ESD in this ex vivo porcine model because simulated lesions were set to be ≥3 cm in diameter. This may have caused the significant difference in treatment outcomes associated with operator experience. Since the OR of the selected ESD method was as high as that of operator experience, P‐ESD was associated with the reduction of ESD difficulties.

The present study had several limitations. First, the ex vivo model had no blood circulation, and the ESD procedures did not include hemostasis for intraoperative bleeding. However, P‐ESD may be effective for hemostasis in bleeding sometimes occurring during injection because the hemostasis procedure can be started immediately after bleeding onset without device replacement. Although the water‐jet system was not used in this study due to the lack of procedure‐related bleeding, this system would be essential in reducing the gap between ESD in a clinical setting and in an ex vivo porcine model. Second, the planned use of ESD‐assisting techniques was restricted. In most cases, the circumferential mucosal incision was completed after partial submucosal dissection to make the mucosal flap but before completion of submucosal dissection. Thus, the study's ESD procedure differs from that of real clinical practice. Third, the amounts and locations of injections were not standardized but depended on each operator's judgment. The number of injections, a secondary outcome, may have been affected by this operator decision. Fourth, this study focused on larger lesions that are associated with ESD difficulties. The efficacy of P‐ESD compared to C‐ESD for standard lesions is still to be confirmed. Finally, this study compared treatment outcomes between P‐ESD and C‐ESD and did not include ESD using other knives with water‐jet injection functions. Further studies comparing the use of ProKnife with other ESD devices in human patients and targeting a wider range of lesions are warranted.

## CONCLUSIONS

This study revealed the efficacy and safety of P‐ESD compared with C‐ESD in an *ex vivo* porcine stomach model. P‐ESD contributed to shortening ESD procedure time with high technical success and safety. The selection of C‐ESD was an independent predictive factor associated with ESD difficulties. A reduction of hyaluronic acid solution used in P‐ESD may contribute to reducing associated medical costs.

## CONFLICT OF INTEREST

Eikichi Ihara participated in the funded research of Takeda Pharmaceutical Co., Ltd. and belongs to the endowed course supported by the companies mentioned, including Ono Pharmaceutical Co., Ltd., Miyarisan Pharmaceutical Co. Ltd., Sanwa Kagaku Kenkyusho Co., Ltd., Otsuka Pharmaceutical Factory, Inc., Fujifilm Medical Co., Ltd., Terumo Corporation, Fancl Corporation, Ohga Pharmacy, and Abbott Japan, LLC. Eikichi Ihara received a lecture fee from Takeda Pharmaceutical Co. Yoshihiro Ogawa is conducting a joint study with Fancl Corporation and Fujifilm Medical Co., Ltd. The other authors declare they have no conflict of interest.

## FUNDING INFORMATION

This study received no funding. Five ProKnife were provided free of charge by Boston Scientific.

## Supporting information


**Table S1**. Background characteristics and outcomes in 10 cases of the pilot study
**Table S2**. Background characteristics and ESD method of each operator

## Data Availability

The authors confirm that the data supporting the findings of this study are available within the article and its supplementary materials.
