# Supplementary material for: Comparisons of outcomes between ProKnife injection endoscopic submucosal dissection and conventional endoscopic submucosal dissection for large gastric lesions in ex vivo porcine model study: A randomized controlled trial
Source: DEN Open. 2022 Jan 26;2(1):e91. doi: 10.1002/deo2.91 (PMC8828196; doi:10.1002/deo2.91)
Supplement: Supplementary file 1 — Table S1. Background characteristics and outcomes in 10 cases of the pilot study Table S2. Background characteristics and ESD method of each operator [file DEO2-2-e91-s001.docx]

**Supplemental table 1. Background characteristics and outcomes in ten cases of the pilot study**

| No | ESD experience | Location | Position | Estimated tumor size, mm | ESD procedure time | No. of injection | Injection volume, mL | Tumor size, mm | Resected specimen size, mm | En bloc resection | Complete resection |
| --- | --- | --- | --- | --- | --- | --- | --- | --- | --- | --- | --- |
| 1 | ≥ 50 cases | U/M | Greater curvature | 35×30 | 25 | 3 | 19 | 33×30 | 38×32 | En bloc | Complete |
| 2 | ≥ 50 cases | L | Posterior wall | 35×30 | 27.5 | 2 | 10 | 36×30 | 48×38 | En bloc | Complete |
| 3 | 0-49 cases | U/M | Anterior wall | 35×30 | 41 | 4 | 24 | 35×25 | 40×30 | En bloc | Complete |
| 4 | 0-49 cases | U/M | Greater curvature | 40×30 | 45 | 5 | 28 | 45×28 | 52×30 | En bloc | Incomplete |
| 5 | 0-49 cases | L | Greater curvature | 35×35 | 85.2 | 6 | 32 | 33×30 | 45×40 | En bloc | Complete |
| 6 | ≥ 50 cases | U/M | Anterior wall | 35×25 | 47 | 3 | 29 | 35×30 | 35×30 | En bloc | Complete |
| 7 | ≥ 50 cases | L | Anterior wall | 35×30 | 38.5 | 4 | 23 | 37×26 | 47×30 | En bloc | Complete |
| 8 | 0-49 cases | L | Posterior wall | 35×30 | 85.1 | 9 | 48 | 30×24 | 35×30 | En bloc | Incomplete |
| 9 | 0-49 cases | L | Posterior wall | 35×35 | 97.2 | 6 | 28 | 34×34 | 44×40 | En bloc | Complete |
| 10 | ≥ 50 cases | U/M | Lessor curvature | 35×30 | 26.3 | 4 | 22 | 31×24 | 42×33 | En bloc | Complete |

ESD; endoscopic submucosal dissection

U; upper third of the stomach, M; middle third of the stomach, L; lower third of the stomach

**Supplemental table 2. Background characteristics and ESD method of each operator**

| Operator | ESD experience | No. of lesions | Location  U or M / L | Position  G / others | Estimated long axis diameter  30-39mm / ≥ 40 mm | ESD method  C-ESD/P-ESD |
| --- | --- | --- | --- | --- | --- | --- |
| 1 | 0-49 cases | 14 | 9 / 5 | 7 / 7 | 11 / 3 | 10 / 4 |
| 2 | 0-49 cases | 9 | 5 / 4 | 5 / 4 | 4 / 5 | 5 / 4 |
| 3 | 0-49 cases | 9 | 4 / 5 | 3 / 6 | 7 / 2 | 4 / 5 |
| 4 | 0-49 cases | 2 | 0 / 2 | 1 / 1 | 2 / 0 | 0 / 2 |
| 5 | 0-49 cases | 1 | 1 / 0 | 0 / 1 | 1 / 0 | 0 / 1 |
| 6 | 0-49 cases | 1 | 0 / 1 | 1 / 0 | 1 / 0 | 0 / 1 |
| 7 | 0-49 cases | 1 | 1 / 0 | 0 / 1 | 1 / 0 | 0 / 1 |
| 8 | ≥ 50 cases | 7 | 3 / 4 | 4 / 3 | 4 / 3 | 4 / 3 |
| 9 | ≥ 50 cases | 7 | 4 / 3 | 1 / 6 | 2 / 5 | 3 / 4 |
| 10 | ≥ 50 cases | 7 | 2 / 5 | 2 / 5 | 6 / 1 | 4 / 3 |
| 11 | ≥ 50 cases | 5 | 5 / 0 | 2 / 3 | 1 / 4 | 2 / 3 |
| 12 | ≥ 50 cases | 5 | 2 / 3 | 3 / 2 | 4 / 1 | 2 / 3 |

ESD; endoscopic submucosal dissection

U; upper third of the stomach, M; middle third of the stomach, L; lower third of the stomach

G; greater curvature, C-ESD, conventional ESD, P-ESD, ProKnife injection ESD
